# Pregnant women’s knowledge of non-pharmacological techniques for pain relief during childbirth

**DOI:** 10.18332/ejm/145235

**Published:** 2022-02-04

**Authors:** Maria A. Heim, Maria Y. Makuch

**Affiliations:** 1 Department of Obstetrics and Gynecology, Faculty of Medical Sciences, State University of Campinas (UNICAMP), Campinas, Brazil; 2 Center for Research in Reproductive Health of Campinas (CEMICAMP), Campinas, Brazil

**Keywords:** non-pharmacological techniques, knowledge, labor, pain, pain relief, childbirth

## Abstract

**INTRODUCTION:**

The objective of the study was to assess knowledge of pregnant women in the third trimester of pregnancy on non-pharmacological techniques for pain relief during labor and childbirth.

**METHODS:**

A cross-sectional study was conducted at a tertiary care facility of the University of Campinas, Brazil. The participants were 171 pregnant women, aged 18–35 years with 36 or more weeks of a singleton pregnancy. Participants responded to a questionnaire with data about sociodemographic and obstetric characteristics, knowledge on non-pharmacological techniques for pain relief during childbirth, the source of the information on these techniques, practice of physical activity and occurrence of pain during pregnancy. Parous women responded on the use of non-pharmacological techniques for pain relief during childbirth in previous deliveries. Multiple regression analysis with stepwise criteria of selection of variables was used to identify variables significantly associated with knowledge of non-pharmacological techniques for pain relief during childbirth.

**RESULTS:**

A total of 165 (96.5%) participants reported knowledge on at least one non-pharmacological technique; 87.1% on the use of a warm shower during labor for pain relief, 80.7% on the use of the birthing ball, and 74.8% on breathing techniques. There were no significant differences between nulliparous and parous women. The main source of information reported was the Internet. Multivariate analysis showed that pregnant women who had pain during pregnancy reported more knowledge on the use of warm showers during labor (OR=2.64; 95% CI: 1.03–6.73).

**CONCLUSIONS:**

Most women had knowledge of at least one non-pharmacological technique for pain relief during childbirth.

## INTRODUCTION

Labor and childbirth pose a physiological and psychological challenge for women. Pain associated with labor and childbirth, described as a physiological condition and as one of the most unique and intense forms of pain that can be experienced, it is intense, expected, and considered a normal event. Each woman has a unique way to cope with childbirth pain. A better comprehension of pain during labor and childbirth is important for the development of strategies to help women during this experience to develop capacity to reduce the use of pharmacological interventions^1-3^. Many factors influence women’s experience of pain associated with labor, including prior experience of delivery and sociocultural factors, such as years of schooling^3^. Fear of childbirth pain in many settings is frequent among women and has been described as one of the main reasons associated with the solicitation of elective caesarean delivery, increasing the possibilities of antepartum depression and anxiety levels^4^.

Non-pharmacological techniques can be used to relieve pain and improve women’s well-being during childbirth. The World Health Organisation^5^ and the Ministry of Health of Brazil^6^ recommend, among other sources for pain relief and to improve laboring women’s well-being, the use of non-pharmacological techniques. Some of these techniques are non-invasive, help women to develop strategies to control pain during labor and childbirth, are safe for the woman and the newborn, do not present side effects, present benefits, and have no cost^7-10^. It has been reported that many women in labor and during childbirth would like to use non-pharmacological techniques of pain relief including massage and heat application, position changes, breathing exercises, bathing in labor, aromatherapy, acupressure and acupuncture, TENS, among others^7,11,12^.

The use of non-pharmacological techniques for pain relief is associated with previous knowledge and adequate guidance that enable women to use them correctly and efficiently for pain relief, anxiety, and to be in control of their labour^13^. It has been reported that in a group of pregnant women with superficial knowledge of the non-pharmacological techniques, the majority (83.3%) did not have clear information regarding what techniques can be used and are adequate for the different stages of labor and childbirth. The technique that pregnant women reported more frequently was breathing exercises (51.8%) followed by massage (36.7%), position changes (32.2%), and relaxation (26.5%)^14,15^. Furthermore, it was reported that women were interested in using these techniques; however, most of the participant women did not know how to use these techniques in a satisfactory manner^15^.

In general, there is scarce information and there are gaps regarding the knowledge on what pregnant women know about non-pharmacological techniques for pain relief during labor and childbirth as well as on the source of the information. Due to this paucity of data, our objectives were to assess the knowledge of pregnant women regarding non-pharmacological techniques for pain relief during labor and childbirth and the source of the information they reported.

## METHODS

### Participants and setting

A cross-sectional study was conducted between April 2019 and March 2020 with pregnant women in the third trimester of pregnancy regarding knowledge of non-pharmacological techniques for pain relief during labor and childbirth, the source of that information, the practice of physical activity during pregnancy, and reported pain during pregnancy. The study was conducted in a teaching referral hospital for women, University of Campinas Faculty of Medical Sciences, Brazil.

The inclusion criteria were: pregnant women aged 18–35 years (to have the possibility to include more primiparous women), with a singleton pregnancy, and with 36 or more weeks of pregnancy. We excluded pregnant women with medical pathologies such as human immunodeficiency virus infection, heart diseases, pregnancy-related pathologies (blood hypertension and/or placental insufficiency), deaf-mute women, and women with two previous caesarean deliveries. All participants were selected according to the inclusion and exclusion criteria the day before their routine antenatal consultation through the medical record system of the hospital and all identified women were invited to participate. All the enrolled women responded to a questionnaire elaborated for this study, which included data about sociodemographic and obstetric characteristics, knowledge on the non-pharmacological techniques for pain relief during labor and childbirth (including maintaining an upright position, using a birthing ball, breathing exercises, relaxation technique, massage, use of water shower, aromatherapy, acupressure, TENS, and continuous support of a companion chosen by the laboring women), the source of information on these techniques, practice of physical activity, and the occurrence of pain during pregnancy. The questionnaire with closed-ended questions was pre-tested for content and comprehension, with 20 pregnant women of the same facility before the initiation of the study. The corrected version was pre-tested with 20 pregnant women at the same site and no further corrections were needed.

### Statistical analysis

For statistical analysis, we used frequency and percentage for categorical variables and mean and standard deviation (SD) for numerical variables. We compared knowledge of the techniques and practice of physical activities using the chi-squared or Fisher’s exact tests (for expected values <5). Subsequently, we used the Mann-Whitney U test because the variables did not present a normal distribution. Among parous women, we also assessed the use of non-pharmacological techniques for pain relief during childbirth in previous deliveries. We performed bivariate and multivariate analyses to compare women’s knowledge of non-pharmacological methods for pain relief with reported pain during pregnancy. The variables included in the analysis regarding the non-pharmacological techniques performed during labor were as follows: maintaining an upright position, using a birthing ball, breathing exercises, relaxation technique, massage, use of water shower, aromatherapy, acupressure, TENS, and continuous support of a companion chosen by the laboring women. The level of significance was set at p<0.05.

## RESULTS

A total of 171 pregnant women were enrolled. The age of the participants (mean ± SD) was 27.7 (± 4.7) years. Moreover, 53 (30.9%) were nulliparous, 121 (70.6%) had 13 or more years of schooling, and 103 (60.2%) were working outside home (Table 1). Regarding pain during pregnancy, 131 (76.6%) reported experiencing pain during pregnancy, 82 (53.2%) had abdominal pain, 60 (38.9%) pelvic pain, and 51 (33.1%) lumbosacral pain. The practice of physical exercise was reported by 49 (28.6%) women, of whom 45 (91.8%) reported that the physical activity performed was walking, 16 (35.5%) said they walked 2 days a week, 14 (31.1%) walked 3 days a week, 14 (31.1%) walked more than 3 days a week, and 5 (11.1%) walked daily.

**Table 1:** Sociodemographic characteristics of the participants (N=171)

*Variable*	*n (%)*
**Age** (years)	
<20	6 (3.5)
20-29	94 (55.0)
30-35	71 (41.5)
**Education years**	
0-4	21 (12.3)
5-9	7 (4.1)
10-12	22 (12.9)
>13	121 (70.7)
**Cohabitation status**	
With a partner	157 (91.8)
**Occupation**	
Housewife	60 (35.1)
Work outside home	103 (60.2)
Other^a^	8 (4.7)
**Number of pregnancies** (including present)	
1	53 (31.0)
2	57 (33.3)
3	39 (22.8)
>4	22 (12.9)
**Previous vaginal deliveries^b^**	
1	48 (28.0)
2	13 (7.6)
>3	5 (2.9)
**Mode of childbirth desired**	
Vaginal	118 (69.0)
Caesarean	37 (21.6)
Not known	16 (9.4)

a Student and unemployed

b Excluded previous caesarean deliveries.

**Figure 1:**
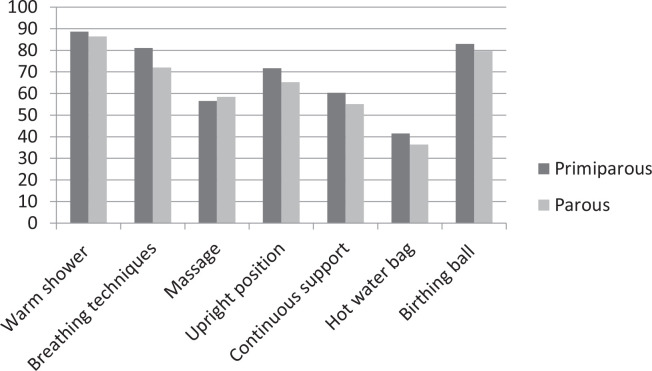
Knowledge of non-pharmacological techniques for pain relief among primiparous and parous women

Regarding knowledge of non-pharmacological techniques for pain relief during labor and childbirth, 165 (96.5%) participants had some knowledge of at least one technique, and 87.1% had information on the use of a warm shower during labor for pain relief, 80.7% on the use of the birthing ball, and 74.8% on the use of breathing exercises (Figure 1), without significant differences between nulliparous and parous women. Regarding where the participants obtained information about non-pharmacological methods for pain relief during labor, the main source reported was the Internet (Table 2). Among the 80 parous women who reported having used non-pharmacological techniques for pain relief during labor and childbirth in previous deliveries, 56.6% had received guidance from healthcare providers on these techniques only during labor. The most frequently used techniques in previous labor and childbirth were: warm showers (37 women), upright position (27 women), birthing ball (22 women), breathing exercises (19 women), massage (16 women); 38 (45.2%) reported they did not use any technique.

**Table 2:** Main sources of information women reported on non-pharmacological techniques for pain control during labor and delivery

*Source of information^a,b^*	*Nulliparous women (n=105)^a^ n*	*Parous women (n=66)^a,b^ n*
Internet	53	54
Relatives	18	20
Reference hospital	9	34
Friends	9	24
No answer	16	-

a More than one option was allowed

b Two women without information.

When we compared the practice of physical activity and the desire for a vaginal or caesarean delivery with knowledge about non-pharmacological methods for pain relief during labor, we did not observe any significant differences. We also did not observe any significant differences in knowledge of non-pharmacological methods for pain relief between nulliparous and parous women. After multivariate analyses, we found that pregnant women with pain during pregnancy reported more knowledge on the use of warm showers during labor for pain relief (OR=2.64; 95% CI: 1.03–6.73, p<0.043) (Table 3).

**Table 3:** Regression analysis for associated pain during pregnancy (N=171)

*Selected variables*	*Categories*	*p*	OR*	*95 % CI*
Knowledge of warm shower	No (Ref.) Yes	0.043	1 2.64	1.03 – 6.73

* OR: odds ratio for pain; (n=40 no; and n=131 yes).

## DISCUSSION

Our results show that most of the pregnant women had some knowledge about non-pharmacological methods for pain relief during labor and childbirth, and the most mentioned techniques were the use of warm showers, the use of the birthing ball and breathing techniques; and the main source of information reported was the Internet. However, the women in our study did not mention the water birth or tub, probably because it is uncommon practice in Brazil and it is not part of the practices offered at our hospital.

Most of our results are consistent with studies conducted during the last decade. A study conducted in Nigeria has shown that 31% of pregnant women did not have knowledge of non-pharmacological methods for pain relief in labor and childbirth^14^, and a study in India reported that 22% of women did not have knowledge of these techniques^13^. However, knowledge on these techniques does not mean that women know how to use them. Considering this, it was reported by Anarado et al.^14^ that 80% of pregnant women were aware that pain during labor could be relieved using non-pharmacological methods, but only one-third was able to identify at least four of these methods, enumerate at least two advantages and disadvantages, and report the self-efficacy of use of these methods.

Previous childbirths have been associated with more knowledge of non-pharmacological techniques for pain relief. It has been reported that the level of knowledge is higher among women with two previous childbirths^2,11,13^; however, we found that previous childbirths were not a good indicator regarding the knowledge women had. This result indicates that previous experience without active guidance or support is not sufficient for women to acquire knowledge and to use non-pharmacological methods correctly in a subsequent childbirth. This may explain why our results did not show significant differences in knowledge among nulliparous and parous women.

It can be supposed that women who desire vaginal delivery, a situation where women’s participation is more active during labor, were more prone to practice physical activities. However, we did not observe this association in our study. Our findings were different from the findings of a recent study that described an association between the practice of physical exercise during pregnancy for at least 6 months, one to three times a week for at least 30 min, and a lower chance of caesarean delivery^16^.

We found that women who experienced pain during pregnancy were those who had more knowledge regarding the use of warm showers during labor. It has been reported that this technique was used by pregnant women to alleviate Braxton-Hicks contractions and promote relaxation. It may be considered that women are familiar with this technique because it is frequent practice that non-pregnant women use this technique to alleviate dysmenorrhoea^17^. Most of the published evidence on the benefits of using water for pain control comes from studies on the use of immersion in water^12^. However, our results even though similar regarding the use of warm water in alleviating pain, refer only to the use of warm showers. This is a relevant issue that deserves more study considering that in several low- and middle-income countries, it is more realistic to think that women have access to a warm shower rather than any form of immersion in water^12^.

Previous studies have shown that pregnant women had scarce knowledge but were interested in some non-pharmacological techniques for pain relief during childbirth. The breathing technique, massage, and upright position^17^ were described by pregnant women as techniques to alleviate discomfort and for pain relief during labour^14^. Our results showed that the recommendations from the Brazilian Ministry of Health^6^ about the use of non-pharmacological techniques, such as breathing exercises, massage and relaxation, and to incentivize women to adopt an upright position during labor to alleviate pain and improve well-being, are not always followed by healthcare providers and healthcare services. Although the recommendations from the Ministry of Health^6^ give much emphasis to these recommendations, it still has not been implemented in all obstetric services, and frequently it is only implemented as guidance and support in the labor ward and scarce information provided during antenatal visits.

We found that the Internet was the main source of information about non-pharmacological techniques for pregnant women. This result was in agreement with previous study which reported that Internet was the main source that women used to improve knowledge regarding these techniques and YouTube videos were described as a resource for this kind of search^18^. We found that almost all the parous and 50% of the nulliparous women reported the Internet as the main source of knowledge, and less than one-fifth of the participants reported that they received the information at the hospital during antenatal consultations or when they were admitted to the hospital in a previous delivery. We need to consider that women who did not attend antenatal education programs and wanted to be prepared for childbirth and postpartum also used this resource to obtain information^19^.

We found that although our assessment was performed in the third trimester of pregnancy, which may indicate that the participants had already attended several antenatal consultations, our results do not indicate that the women had received guidance on the use of non-pharmacological techniques for pain relief.

Our results show that healthcare professionals providing outpatient obstetric care must not only offer good quality of care for pregnancy control but also include strategies to include guidance on non-pharmacological techniques for pain relief. Considering that the participants identified the Internet as the main source of information about the non-pharmacological techniques for pain relief, it is important to conduct more studies that will contribute to the elaboration of adequate messages. The transmission of this type of information to pregnant women in an easy-to-understand manner is important and will help them become familiar with these methods. It would be interesting to include this kind of information on the website of healthcare facilities and create channels of interaction for the users with the healthcare professionals of the maternity hospitals were they receive antenatal care and receive attention during childbirth. It is desirable that maternity facilities offer some training on non-pharmacological techniques for pain relief to healthcare providers who provide assistance to pregnant women.

### Strengths and limitations

The strength of our study was the number of participating women. However, a limitation is that the study was conducted in only one teaching referral hospital.

## CONCLUSIONS

Most of the interviewed women had some knowledge of at least one non-pharmacological technique for pain relief during labor and childbirth. The most mentioned techniques were use of a warm shower and use of birthing balls and breathing techniques. The main source of information was the Internet.

## Data Availability

The data supporting this research are available from the authors on reasonable request.
