# Correction: Pregnant women’s knowledge of non-pharmacological techniques for pain relief during childbirth

**DOI:** 10.18332/ejm/147248

**Published:** 2022-03-24

**Authors:** European Publishing

**Affiliations:** 1 European Publishing, Heraklion, Greece

**Keywords:** non-pharmacological techniques, knowledge, labor, pain, pain relief, childbirth

**Correction on:**

**Pregnant women’s knowledge of non-pharmacological techniques for pain relief during childbirth**

**By Maria A. Heim, Maria Y. Makuch**

**European Journal of Midwifery, Volume 6, Issue February, Pages 1-6**

**Publish date: 7 February 2022**

**DOI: https://doi.org/10.18332/ejm/145235**

In the original article, in the pdf file of the manuscript, the article DOI number did not correspond to the assigned DOI number. The correct manuscript DOI is https://doi.org/10.18332/ejm/145235. The pdf file has been replaced.

